# Hepatic Portal Venous Gas with Pneumatosis Intestinalis

**DOI:** 10.4021/gr2009.01.1258

**Published:** 2009-01-20

**Authors:** Nayan M. Patel

**Affiliations:** Banner Good Samaritan Medical Center, Liver Disease Center, 1300 N. 12th Street, Suite 507, Phoenix AZ 85006, USA

**Keywords:** Pneumatosis intestinalis, Esenteric ischemia, Hepatic portal venous gas

## Abstract

Hepatic portal venous gas with pneumatosis intestinalis are radiological clues of intestinal ischemia. Prompt evaluation and a high index of clinical suspicion for the diagnosis of acute mesenteric ischemia are necessary given the high mortality of this condition. We present a case of mesenteric ischemia, radiological clues to diagnosis, and associated mechanisms.

## Case report

A 77 year old male with the past medical history of diabetes mellitus type 2 complicated by a right BKA, hypertension, hypercholesterolemia, and chronic kidney disease presents to the ED for shortness of breath and abdominal pain. Three days prior to admission he noted a sudden onset of crampy and aching pain across his lower abdomen, constant in nature, relieved somewhat with over-the-counter Maalox and Pepcid, but never completely subsiding. The same evening the pain started, he experienced nausea, but no vomiting. He had no history of biliary colic, or abdominal angina. The patient also noted increasing dyspnea, and was found sitting on the edge of his bed trying to catch his breath. He denied any recent fever or chills, hematochezia, or melena. In the ED, he was afebrile with a BP 101/50 mmHg, pulse of 93/min, and oxygen saturation of 92% on 2 liters nasal cannula, and respirations of 24/min. His cardiac exam was regular. His lungs revealed bibasilar crackles. His abdomen exam revealed an obese abdomen with some pain to deep palpation in the lower quadrants, but overall fairly benign. Bowel sounds were normoactive, and there was no hepatosplenomegaly. Rectal exam was guaic negative without any masses.

Laboratory data was significant for a creatinine 3.6 mg/dl, with normal liver function tests and coagulation tests. A V/Q scan was read as high probability for pulmonary embolism, later revised to intermediate probability. He was admitted to the hospital for treatment with IV unfractionated heparin, with the diagnosis of pulmonary embolism. His abdominal pain was never clarified on admission, but was still concerning and a CT scan of the abdomen without contrast because of renal insufficiency was ordered in the morning.

**Figure 1:**
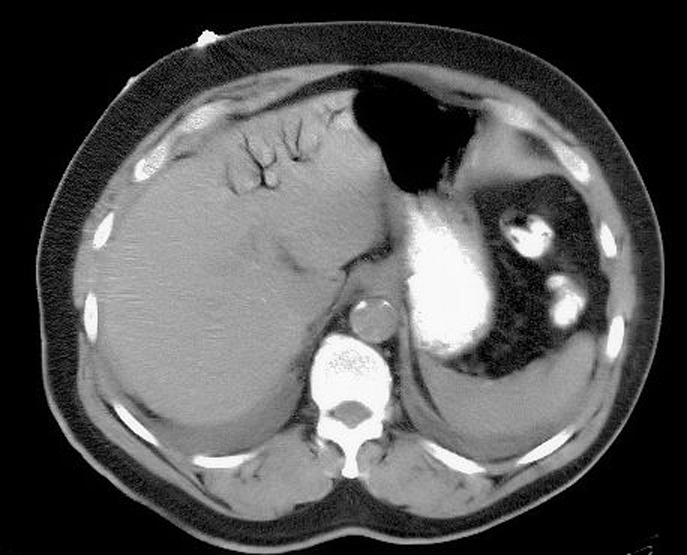
Showing portal venous air in the left hepatic lobe.

**Figure 2:**
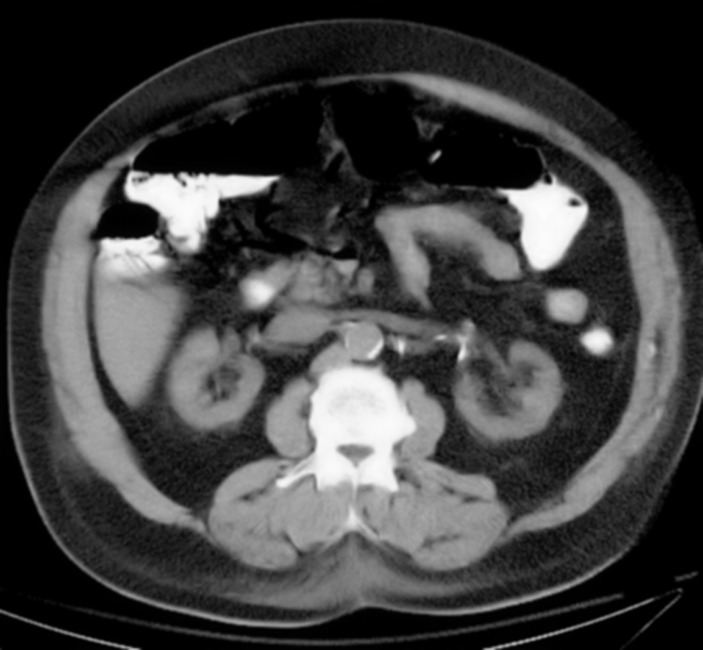
Showing mesenteric venous air throughout much of the right-sided mesenteric system and an air/blood level within the SMV.

**Figure 3:**
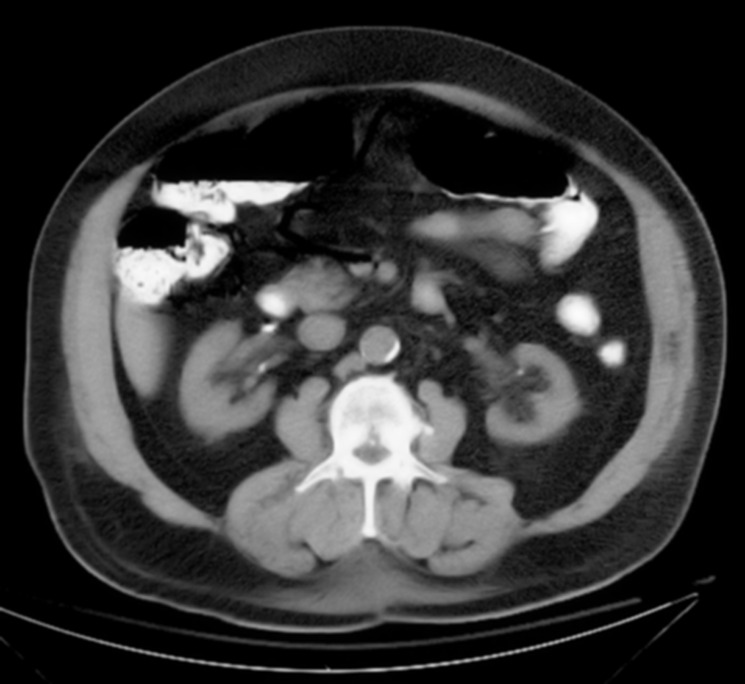
Showing pneumatosis of the right hemicolon.

Figure 1 shows portal venous air in the left hepatic lobe. Figure 2 shows mesenteric venous air throughout much of the right-sided mesenteric system and an air/blood level within the SMV.Figure 3 shows pneumatosis of the right hemicolon, findings consistent with acute mesenteric ischemia. STAT surgical consultation was obtained and after discussion with the family regarding the high operative risk of mortality versus certain sepsis and mortality without surgery, the patient and family decided to become comfort care. The patient’s pain was controlled, and comfort measures were initiated with his family at the bedside. The patient ultimately demised after two days. A higher clinical suspicion may have led to earlier diagnosis.

## Discussion

Hepatic portal venous gas (HPVG), as well as pneumatosis intestinalis (PI), are radiological clues and not diagnoses. Two groups should be distinguished at the time of diagnosis, those who have HPVG with associated PI, and those who have HPVG without associated PI. When associated with PI, the cause is usually an intestinal ischemia event, and in a symptomatic patient it justifies performing systematic exploratory surgery [1]. In a review of 182 cases, HPVG was most commonly due to bowel necrosis (43%), digestive tract dilatation (12%), intraperitoneal abscess (11%), ulcerative colitis (4%), gastric ulcer (4%), Crohn disease (4%), complications of endoscopic procedures (4%), intraperitoneal tumor (3%), and other (15%). The overall mortality was 39%, but varied depending on the underlying disease. The mortality was high in cases of HPVG associated with bowel necrosis (75%). On the other hand, there were no lethal cases of HPVG associated with ulcerative colitis, intraperitoneal tumor, Crohn disease, cholangitis, pancreatitis, or complications of endoscopic procedures [2].

Gas in the portal venous system is carried by the centrifugal flow of blood in the periphery of the liver, appearing to extend within 2 cm of the hepatic capsule, while gas in the biliary tract moves within the centripetal flow of bile, thus appearing more centrally in the liver. Therefore, a history of biliodigestive anastomosis, endoscopic papillotomy, biliary endoprosthesis, or choledocointestinal fistula are important in the differential diagnosis [1].

Speculative mechanisms proposed for this finding include bowel distension, presence of mucosal defects, and sepsis. HPVG can be visualized by conventional radiography, ultrasound, and CT. Radiographic criteria for diagnosis include a branching radio-lucency extending to 2 cm within the liver capsule on abdominal x-ray or CT [3]. X-rays are most revealing when taken with the patient in the left lateral decubitus position. Sonographic features include either high echogenic particles flowing in the portal vein or poorly defined, high echogenic patches within the hepatic parenchyma [4]. CT and sonography are more sensitive than conventional radiography to diagnose HPVG [5].

In the absence of a clinical indication for emergency laparotomy, mesenteric angiography remains the investigation of choice in suspected acute mesenteric ischemia. Early angiography has been shown to improve survival rates. Mesenteric angiography can usually differentiate embolic from thrombotic arterial occlusions. Emboli usually lodge where the artery tapers, which is just after the first major branch of the SMA-the middle colic artery. In contrast, thrombotic disease usually involves the origin of the SMA [6].

Acute mesenteric ischemia can be categorized into 4 specific types: arterial embolic, acute mesenteric thrombosis, non-occlusive mesenteric ischemia, and mesenteric venous thrombosis. Arterial emboli are the predominant cause of acute mesenteric ischemia accounting for approximately 40% to 50% of cases. The majority of emboli originate from a cardiac source. Acute mesenteric thrombosis accounts for 25% to 30% of all events. Mesenteric ischemia due to arterial thrombosis occurs in the setting of severe atherosclerosis, with the most common site near the origin of the SMA. Patients with SMA embolism or thrombosis typically have an acute onset of symptoms and rapid deterioration. With an embolism, the onset of symptoms is usually dramatic because of lack of collateral circulation, and it manifests as severe and unrelenting abdominal pain, nausea, vomiting, and urgent bowel evacuation. This is the classical abdominal pain out of proportion to physical findings. Patients with SMA thrombosis frequently report a prodromal symptom complex of postprandial pain, nausea, and weight loss associated with chronic intestinal insufficiency [6].

Approximately 20% of patients with mesenteric ischemia have nonocclusive disease, a poorly understood entity that often includes low cardiac output and diffuse mesenteric vasoconstriction. It most frequently occurs in the setting of an acute hemodynamic insult in elderly and critically ill patients, and in those with severe mesenteric atherosclerosis. Mesenteric venous thrombosis is the least common cause of mesenteric ischemia, representing up to 10% of all patients. Most cases are thought to be secondary to other intra-abdominal pathologic conditions (such as malignancy, intra-abdominal sepsis, or pancreatitis), or clotting disorders [6].

In regards to mortality, the etiology of mesenteric ischemia does make a difference. In a review of over 3000 patients, the mortality rate following surgical treatment of arterial embolism and venous thrombosis (54.1% and 32.1%) was less than that after surgery for arterial thrombosis and non-occlusive ischemia (77.4% and 72.7%) [7]. The clinical suspicion for acute mesenteric ischemia should be high, because in the face of such high mortality, early diagnosis allows for earlier intervention.
